# Dural arteriovenous fistula and sinus thrombosis presenting as parkinsonism and dementia: a case report with literature review

**DOI:** 10.3389/fneur.2024.1336593

**Published:** 2024-03-27

**Authors:** Ranran Tu, Qihua Chen, Lixia Qin

**Affiliations:** ^1^Department of Neurology, The Second Xiangya Hospital, Central South University, Changsha, Hunan, China; ^2^China National Clinical Research Center on Mental Disorders, Changsha, China

**Keywords:** DAVF, parkinsonism, dementia, CVST, Wernicke’s encephalopathy

## Abstract

**Introduction:**

Dural arteriovenous fistula (DAVF) is an uncommon malformation involving an abnormal connection between dural arteries, or the pachymeningeal branches of cerebral arteries, and dural veins. Its exact pathogenesis remains elusive. Known potential triggers for DAVF include cerebral venous sinus thrombosis (CVST), trauma, ear infections, and cranial surgeries. Due to its rarity and diverse clinical presentations, diagnosing DAVF can be a challenge.

**Case description:**

We present a case of DAVF associated with CVST, manifesting as rapidly advancing parkinsonism accompanied by dementia over a month. Brain magnetic resonance imaging (MRI) revealed bilateral symmetric T2 hyperintensities in the basal ganglia and brain stem. Cerebral angiography further confirmed a fistula between the torcular herophili and the transverse-sigmoid sinuses. Despite strong recommendations for transvenous embolization of the fistula, the patient declined the procedure. The anticoagulant therapy and symptomatic treatments administered did not yield any improvement in the patient’s condition. Additionally, we reviewed 27 DAVF-derived parkinsonism and dementia cases.

**Conclusion:**

DAVF must be considered in the differential diagnosis of cases of rapidly progressive parkinsonism with concurrent dementia. Given its potential for treatment and reversibility, timely diagnosis and intervention for DAVF are paramount.

## 1 Introduction

Dural arteriovenous fistula (DAVF) is a rare vascular anomaly characterized by an abnormal connection between dural arteries, or pachymeningeal branches of cerebral arteries, and dural veins. Typically manifesting later in life, DAVF can be triggered by factors such as cerebral venous sinus thrombosis (CVST), trauma, ear infections, and cranial surgeries (1). It is believed that these factors can lead to venous thrombosis. DAVF patients may exhibit a range of symptoms, from pulsatile tinnitus and ophthalmoplegia to acute confusion, cognitive impairment, parkinsonism, and specific neurological deficits. The clinical and radiographic presentations are largely determined by the pattern of venous drainage. The primary treatment approach is endovascular embolization, but surgical intervention and stereotactic radiosurgery serve as viable alternatives (1). Given that DAVF is treatable and many symptoms are reversible, early diagnosis and intervention can significantly enhance a patient’s prognosis and reduce long-term disabilities. In this context, we present a unique case of a patient with rapidly progressing parkinsonism and dementia attributable to DAVF who was misdiagnosed as having Wernicke’s encephalopathy.

### 1.1 Case description

A 59-year-old man came in with worsening and consistent gait difficulties and signs of dementia over the past month. He had not experienced any headaches, double vision, nausea, vomiting, or delirium. His medical history was mostly unremarkable, except for a head injury approximately 1 year ago. Additionally, he had a long history of alcohol consumption. He was initially evaluated at a local hospital, where brain magnetic resonance imaging (MRI) revealed bilateral symmetrical lesions in the brain stem and basal ganglia. Due to the patient’s prolonged history of alcohol abuse and progressive cognitive decline, Wernicke’s encephalopathy was initially suspected at the previous hospital. The patient received high-dose thiamin (300 mg/day, administered via intramuscular injection) for 2 weeks. However, his condition continued to deteriorate, leading to his referral to our neurology department.

Upon examination, he took short steps, showed symmetrical rigidity in all four limbs, general slowness of movement, with mild increased muscle tone and normal strength, hyperreflexia, diminished speech, a soft voice, and cognitive decline without lamination (scoring 18/30 on the Mini-Mental State Examination, especially in attention, calculation, and execution ability). He also exhibited urinary incontinence, and a vascular murmur was audible behind his ears. Laboratory tests, including those for blood count, infections, coagulation function, protein C and S levels, lupus anticoagulant, autoimmune markers (anti-neuronal antibodies, anti-nuclear antibodies, antibodies to extractable nuclear antigen, anti-neutrophil cytoplasmic antibody, anti-cardiolipin antibodies, ds-DNA antibodies, and autoimmune encephalitis antibodies), tumor markers, thromboelastogram, anti-aquaporin 4 antibody, anti-myelin oligodendrocyte glycoprotein antibody, anti-glial fibrillary acidic protein antibody, oligoclonal bands, and metabolic screens, all returned normal results. His blood D-dimer level was measured at 0.73 μg/mL (0–0.55 μg/mL). A lumbar puncture showed that the cerebrospinal fluid pressure exceeded 400mmH2O. Analysis of this fluid indicated normal white cell counts, glucose, and chloride levels, with a slightly elevated protein level of 592.3 mg/L. There were no signs of infection, inflammation, or bleeding.

**Figure 1:**
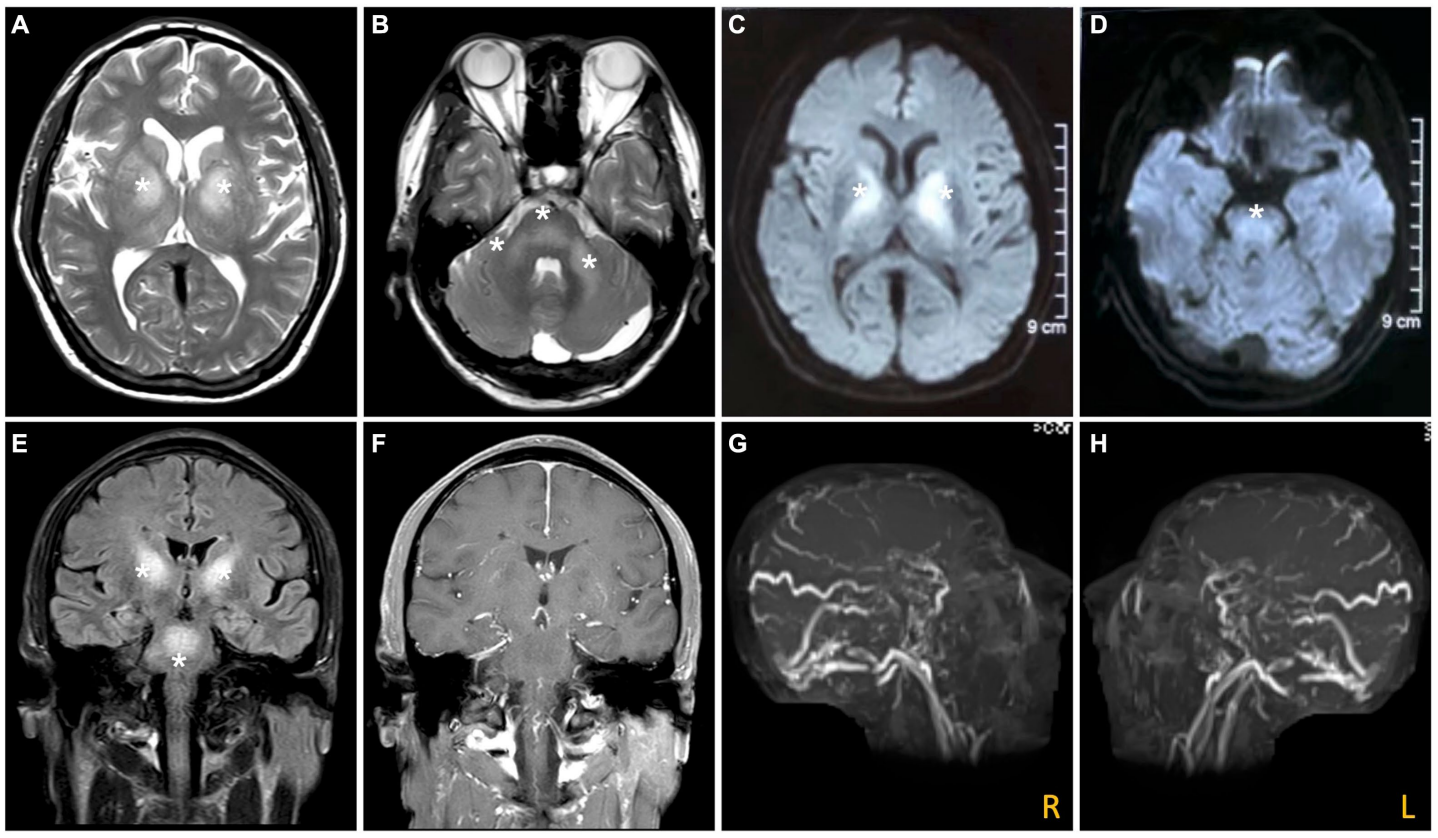
Brain MRI and MRV. The axial section of the T2-weighted image **(A,B)** and the diffusion-weighted image **(C,D)** reveal bilateral symmetrical hyper-intensities in the basal ganglia and brain stem (white asterisk). The lesions present with hyperintensity on fluid attenuated inversion recovery (FLAIR) images **(E)**, and no enhancement on enhancing MRI **(F)**. The MRV **(G,H)** indicates a filling defect in the right transverse, left sigmoid, and superior sagittal sinuses.

**Figure 2:**
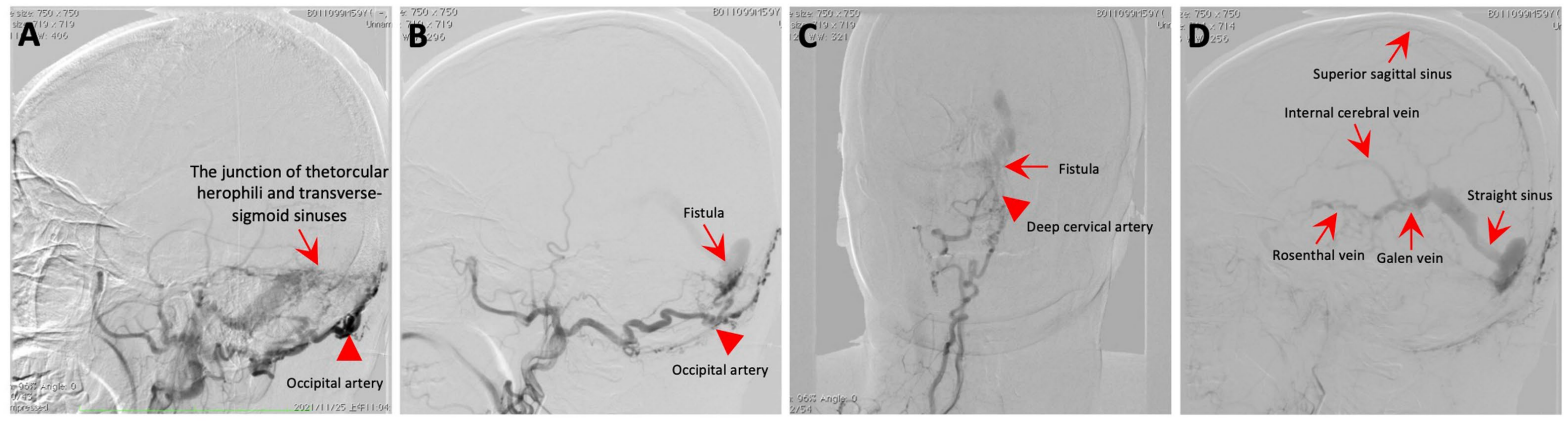
Cerebral angiography. **(A–C)** The angiogram showcases a fistula (red arrow) connecting the torcular herophili to the transverse-sigmoid sinuses, with blood supply from the occipital artery (red arrowhead) and the deep cervical artery (red arrowhead). **(D)** The DAVF drained into the straight sinus, which flowed in a retrograde direction into the vein of Galen, as well as into the internal cerebral veins and basal vein of Rosenthal.

**Figure 3:**
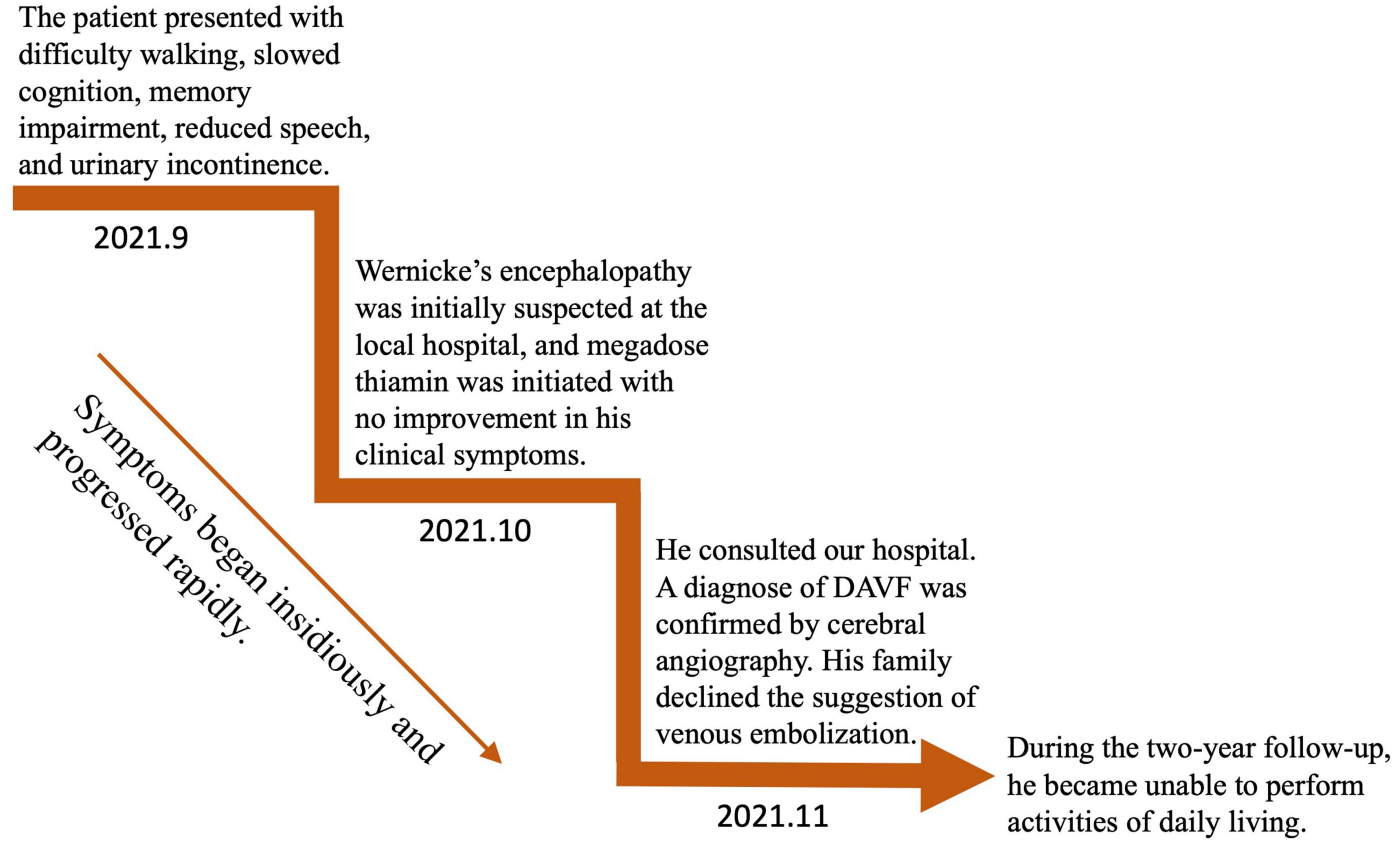
Time course of symptoms, diagnosis, and treatment.

An MRI of the brain revealed bilateral symmetrical lesions in areas such as the pontine, pontine arms, and basal ganglia. These lesions showed restricted diffusion on diffusion-weighted imaging and no enhancement (as seen in Figures 1A–F). Additionally, magnetic resonance venography (MRV) highlighted dilated veins and a filling defect, pointing to possible sinus thrombosis in the superior sagittal sinus (as seen in Figures 1G,H). Cerebral angiography further confirmed thrombosis in the vein of Galen and identified a DAVF between the torcular herophili and transverse-sigmoid sinuses, fed by the occipital artery and arteria cervicalis profunda (as shown in Figure 2). The DAVF drained into the straight sinus, which flowed in a retrograde direction into the vein of Galen, as well as into the internal cerebral veins and basal vein of Rosenthal. Given that DAVFs are treatable and their symptoms can be reversed, it was strongly recommended that the patient undergo transvenous embolization of the fistula. However, the son of the patient declined for personal reasons. Even with subcutaneous injections of low molecular weight heparin (5000AXaIU) every 12 h and symptomatic treatments, including levodopa, there was no improvement in his clinical symptoms. The time course of symptoms, diagnosis, and treatment is shown in Figure 3.

## 2 Discussion

DAVF refers to abnormal shunts between dural arteries and structures such as dural venous sinuses, meningeal veins, or cortical veins. The exact cause of DAVF remains elusive. While many cases appear without any known cause (idiopathic), there are reports suggesting links to CVST, trauma, ear infections, venous hypertension, and prior cranial surgeries. The co-occurrence of DAVF with CVST is relatively rare (2, 3). In our study, we document a case where DAVF is seen alongside CVST. DAVF leads to retrograde blood flow into the straight sinus and the vein of Galen. Thrombosis in the straight sinus and the vein of Galen further exacerbates venous pressure, causing reflux obstruction and insufficient perfusion, subsequently leading to infarction in the corresponding drainage area of brain tissue. Actually, the exact relationship between CVST and DAVF is not well-defined, leading to debates about whether DAVF precipitates CVST or emerges as a consequence of it. Sinus thrombosis can increase venous pressure, which might trigger the development of DAVF by activating dural arteriovenous pathways. This potentially can lead to ischemia because of backward venous flow and blood stagnation. Additionally, the turbulent blood flow observed in DAVF might contribute to thrombus formation (3).

Clinical manifestations linked to DAVF vary widely and are influenced by the pattern of venous drainage. These can range from pulsatile tinnitus and ophthalmoplegia to acute confusion, rapidly advancing dementia, parkinsonism, seizures, cerebellar symptoms, and specific neurological deficits. Notably, DAVF patients with cortical venous drainage and venous ectasia are at a heightened risk of hemorrhage (4). Instances of DAVF patients exhibiting parkinsonism, with or without cognitive dysfunction, are infrequent. Research indicates that parkinsonism in DAVF patients tends to be more prevalent in older male patients, averaging approximately 63 years of age, with cases spanning from 40 to 81 years. The time from initial symptoms to DAVF diagnosis has been documented to range between 1 week and 3 years (5). In this study, we reviewed 27 DAVF patients who have parkinsonism with cognitive dysfunction (Table 1). Of these patients, 18 were male, with an average age of 65 years, ranging from 40 to 87 years old. While the precise mechanism remains unclear, the prevailing theory is that reduced blood flow in the frontal lobes and a perfusion defect in the basal ganglia may be contributing factors to the onset of DAVF-related parkinsonism (5, 7). There is growing evidence suggesting a specific vascular mechanism whereby thalamic damage leads to subacute encephalopathy or swiftly progressing dementia (25). In the case we observed, cognitive impairments included slowed thinking, difficulties with calculations and concentration, memory loss, executive dysfunction, and diminished speech, suggesting dysfunction in the basal ganglia or frontal lobe.

**Table 1:** Cases with DAVF presenting with progressive dementia and parkinsonism.

No.	Author (Year)	Age/gender	Neurologic signs (except dementia and parkinsonism)	CT/MR imaging change	Angiography location/drainage	Treatment	Outcome
1	Mastsuda S. et al. (6)	55/M	Headache	White matter, left thalamus	Right sigmoid sinus/superior sagittal sinus, straight sinus	TAE	Improved
2	Mastsuda S. et al. (6)	78/M	Blurred vision	White matter, subarachnoid space	Right sigmoid sinus/superior sagittal sinus, straight sinus, frontal cortical veins	TAE	Improved
3	Mastsuda S. et al. (6)	69/F	Tinnitus, headache	White matter, subarachnoid space	Left sigmoid sinus/transverse sinus, superior sagittal sinus, straight sinus, cortical veins	TAE	Unimproved
4	Lee PH. et al. (7)	60/F	Normal	White matter	Left transverse-sigmoid sinus/superior sagittal sinus, cortical venous	TAE	Improved
5	Chan HY. et al. (8)	77/M	Double incontinence	Left temporal and occipital lobes	Transverse-sigmoid sinus/vein of Labbe and other cortical veins	TAE	Improved
6	Kajitsni M. et al. (9)	75/M	Transient loss of consciousness after slow deep breathing	Frontal lobes and basal ganglia	Transverse-sigmoid sinus/superior sagittal sinus	TAE	Improved
7	Miura S. et al. (10)	65/M	Diplopia, tinnitus, ataxia	Basal ganglia, white matter	Left transverse-sigmoid sinus/superior sagittal sinus, straight sinus	TAE	Improved
8	Norgueira RG. et al. (11)	79/M	Tinnitus, hearing loss, vertigo	Serpiginous flow voids on the right mesial temporal lobe	Left transverse sinus/ superior sagittal vein, straight sinus, basal vein of Rosenthal	TAE, surgery, and TVE	Improved
9	Netravathi M. et al. (12)	54/M	Headache	Thalamus, globus pallidus, basal ganglia, white matter	Torcula/straight sinus	TAE	Minimal change
10	Netravathi M. et al. (12)	40/M	Urinary incontinence	Gray and white matter	Superior sagittal sinus/ retrograde sinus flow and cortical venous reflux	Unsuccessful TAE and TVE	Deteriorated
11	Shahar T. et al. (13)	59/M	Limitation in right and up-gaze, vertical nystagmus on up-gaze	Right occipital lobe, lenticular nuclei	Straight sinus/vein of Galen, vein of Rosenthal, internal cerebral veins	TAE	Improved
12	Hattori T. et al. (14)	52/F	General fatigue, urinary incontinence, emesis, tinnitus	Whiter matter, basal ganglia	Transverse-sigmoid sinus/superior sagittal sinus, straight sinus	TVE	Improved
13	Fujii H. et al. (5)	69/M	Normal	Frontal lobes, basal ganglia	Superior sagittal sinus/ND	TVE	Improved
14	Jagtap SA. et al. (15)	73/F	Myoclonic jerks	Transverse sigmoid junction	bilateral transverse sinus-sigmoid sinus junction/ND	No	Died
15	Luo Y. et al. (16)	54/M	Urinary incontinence	Flow void clusters at the inner part of the left temporal lobe	Right transverse-sigmoid sinus/ straight sinus, the right transverse sinus	No treatment	No change
16	Luo Y. et al. (16)	75/M	Headache	Dilated vein on the left temporal cortex	Left transverse-sigmoid sinus/straight sinus, left temporal cortical veins	No treatment	Died
17	Ma C. et al. (17)	62/M	Weakness, apathy, urinary incontinence	Normal	Left temporal region/superior sagittal sinus	TAE	Improved
18	Enofe I. et al. (18)	82/F	Seizures	White matter	Transverse sinus/torcular Herophili, superior sagittal sinus, and transverse-sigmoid sinuses	TAE	Improved
19	Lai J. et al. (19)	62/M	Headache, apathy, disorientation, visual hallucinations, generalized status epilepticus	White matter	Right transverse, sigmoid sinus, torcular sinuses, superior sagittal sinus/straight sinus, vein of Galen, pterygoid plexus, cortical veins	TVE	Improved
20	Lai J. et al. (19)	65/F	Tinnitus, headache, visual and auditory hallucinations, ataxia, myoclonus	White matter	Right transverse-sigmoid sinus, right Sigmoid sinus/left transverse sinus, straight sinus, vein of Galen	TVE	Improved
21	Pu J. et al. (20)	51/M	Normal	Lenticular nuclei, white matter	Straight sinus/vein of Galen, vein of Rosenthal, internal cerebral veins	TAE	Improved
22	Gopinath M. et al. (21)	45/F	Normal	White matter	Torcular sinus, superior sagittal sinus/ND	TAE	Improved
23	Tominaga A. et al. (22)	87/F	Depression, disturbance of consciousness	Brainstem, left cerebellar peduncle	left transverse sinus/left superior petrosal sinus	TAE	Improved
24	Prosperini L. et al. (23)	84/M	Delirium, poor speech fluency	White matter	Left transverse sinus/left occipital artery	TAE	Improved
25	Xie J. et al. (24)	70/F	Normal	White matter	Upper and lower sagittal sinuses, bilateral transverse sinuses/ND	No treatment	Deteriorated
26	Xie J. et al. (24)	67/M	Pain and discomfort in the occiput and back of the neck, increased lethargy	Thalamus	Internal cerebral vein, vein of Galen, straight sinus/ND	No treatment	Died
27	Present case	59/M	Normal	Brain stem, basal ganglia	Transverse-sigmoid sinuses/straight sinus, vein of Galen, vein of internal cerebral, vein of Rosenthal	No treatment	Deteriorated

F, female; M, male; TAE, Transarterial embolization; TVE, Transvenous embolization; ND, Not described.

**Figure 4:**
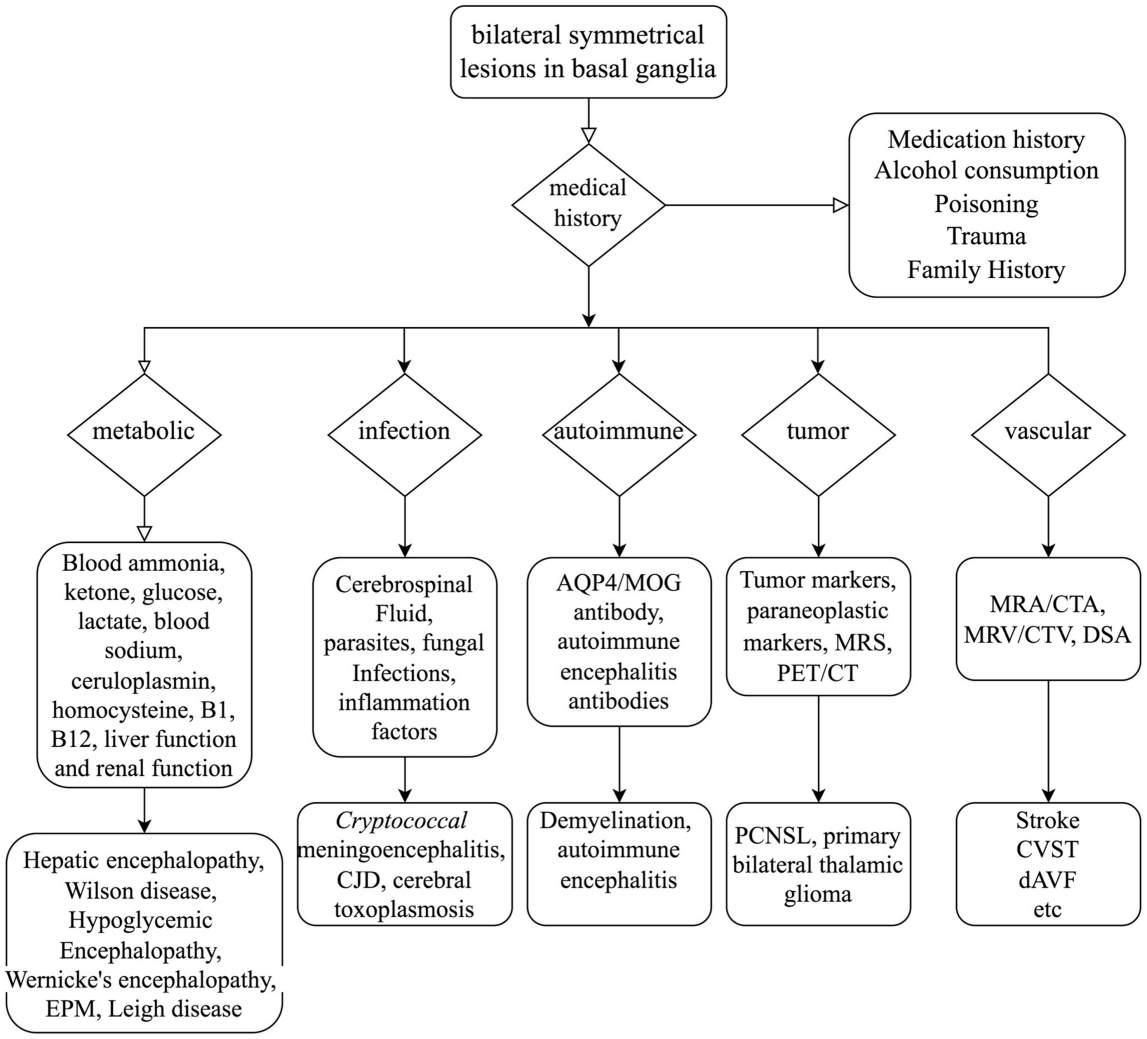
Mind flow chart for the differential diagnosis of bilateral symmetrical lesions in the basal ganglia. AQP4, Aquaporin-4; MOG, myelin oligodendrocyte glycoprotein; MRS, magnetic resonance spectroscopy; PET/CT, positron emission tomography/computed tomography; MRA, magnetic resonance angiography; CTA, computed tomography angiography; MRV, magnetic resonance venography; CTV, computed tomography venography; DSA, digital subtraction angiography; EPM, extrapontine myelinolysis; CJD, Creutzfeldt–Jakob disease; PCNSL, primary lymphomas of the central nervous system; CVST, cerebral venous sinus thrombosis; dAVF, dural arteriovenous fistula.

Given the patient’s rapidly advancing parkinsonism and dementia, combined with a long history of alcohol abuse and bilateral symmetrical lesions in the brain stem and basal ganglia as revealed by MRI, various conditions such as Wernicke’s encephalopathy, central pontine myelinolysis, extrapontine myelinolysis, hepatic encephalopathy, Creutzfeldt–Jakob disease, and glioma must be considered (3). Extensive diagnostic efforts were made to rule out these alternatives (as seen in Figure 4). Vitamin B1 levels were not checked because the patient had already been receiving high doses of thiamine for several days. Wernicke’s encephalopathy is more likely to involve the medial thalamus, hypothalamus, periaqueductal gray matter, and the areas surrounding the third and fourth ventricles on MRI. However, the patient does not present with oculomotor paralysis or psychiatric symptoms, and high-dose thiamine treatment has been ineffective, which does not support the diagnosis of Wernicke’s encephalopathy. Extrapontine myelinolysis is more likely to affect the bilateral striatum, and clinical symptoms often outweigh the radiological changes. Additionally, there is no history of electrolyte disturbances in the patient. Non-invasive vascular imaging techniques, including CT and MRI, could offer clues about the presence of DAVF. In this case, retrograde venous drainage can lead to increased venous pressure, resulting in early vascular-origin edema, which may progress to venous infarction characterized by cytotoxic edema. The MRI lesions primarily concentrate in the basal ganglia region, with relatively preserved midbrain areas, correlating with the susceptibility of deep brain tissues to restricted outflow. Moreover, we acknowledge that, in our case, it cannot be conclusively determined whether the involvement of the deep pontine is caused by the known venous thrombus. Cerebral angiography assists in identifying the arterial feeders and venous outflow of the fistula. The Cognard classification system, commonly utilized to categorize DAVF, separates it into five types based on venous drainage patterns (26). In this particular case, according to the angiograph, the DAVF drains into the straight sinus, with filling of the Galen, internal cerebral, and Rosenthal veins, without cortical venous drainage, unveiling a Cognard type IIa DAVF. However, in this classification, there is no detailed subtyping for reflux into deep veins. In future classifications, refinement based on deep venous drainage may have a more positive significance for prognosis assessment and surgical selection.

In the management of non-Parkinson’s disease (PD) patients with parkinsonism, the treatment involves removing precipitating factors, managing the underlying primary disease, and additionally administering medications used in the treatment of PD, such as amantadine, levodopa, dopamine receptor agonists, monoamine oxidase-B inhibitors, and catechol-O-methyl transferase inhibitors, among others. However, these medications may not provide significant improvement in symptoms. Indeed, damage due to edema or ischemia could be reversible. Early intervention through endovascular embolization, aiming for complete obliteration of the fistula, is highly recommended for DAVF patients. Prior research has shown that the vast majority of DAVF patients experienced significant symptom alleviation, with some even achieving total symptom resolution after undergoing endovascular embolization treatments (27). Moreover, clinical improvement correlates with radiographic improvement. Venous sinus thrombosis may further increase venous pressure. Therefore, in patients without surgery, anticoagulant therapy may potentially reduce venous pressure and thus exert a protective effect. However, the role of anticoagulation in untreated DAVF combined with thrombosis remains unclear. In our case, the patient’s family opted against transvenous embolization of the fistula. Consequently, despite receiving anticoagulant therapy and symptomatic treatments, the patient showed no clinical improvement.

We recognize that our case report has certain limitations. First, despite conducting cerebral angiography, we cannot definitively confirm a causal relationship, as the patient did not undergo treatment for the malformation, followed by recovery. Second, our report is limited to a single case. It is important that future reports include a series of cases to shed light on the relationship between CVST and DAVF. Additionally, functional experiments should be conducted to better understand the underlying mechanisms.

In conclusion, we present a classic case of DAVF characterized by rapidly progressing parkinsonism accompanied by dementia. Cerebral angiography was instrumental in identifying the arterial feeders and venous outflow of the fistula. It is crucial to consider DAVF as a potential underlying cause for rapidly progressing parkinsonism with dementia. As the condition is treatable and its effects potentially reversible, prompt diagnosis and intervention for DAVF are of paramount importance.

### 2.1 Patient perspective

The son of the patient says, “I noticed that over a month ago, my father began to walk slowly, hunching over with a diminished stride, taking small, shuffling steps. Additionally, he has become less responsive, his voice has lowered, and he’s lost control of his bladder and bowels. We sought medical attention at our local hospital and underwent some tests. Given my father’s long history of alcohol consumption, the local hospital treated him for alcohol poisoning. However, not only did his symptoms not improve, they actually worsened. We then consulted the Department of Neurology at the Second Xiangya Hospital. After a cerebral angiogram, the doctor diagnosed him with DAVF and recommended surgery. But the operation can be complicated. We worried about the safety of the surgery as well as a considerable expense, so we could not proceed with the operation.”

## Data availability statement

The datasets presented in this article are not readily available because of ethical and privacy restrictions. Requests to access the datasets should be directed to the corresponding author.

## Ethics statement

Ethical review and approval was not required for the study on human participants in accordance with the local legislation and institutional requirements. Written informed consent from the patients/participants or patients/participants' legal guardian/next of kin was not required to participate in this study in accordance with the national legislation and the institutional requirements. Written informed consent was obtained from the individual(s) for the publication of any potentially identifiable images or data included in this article.

## Author contributions

RT: Writing – original draft, Writing – review & editing. QC: Writing – review & editing. LQ: Writing – original draft, Writing – review & editing.
